# Conceptualizing Productive Engagement in a System Dynamics Framework

**DOI:** 10.1093/geroni/igx018

**Published:** 2017-09-30

**Authors:** Nancy Morrow-Howell, Cal J Halvorsen, Peter Hovmand, Carmen Lee, Ellis Ballard

**Affiliations:** 1 Brown School of Social Work, Center for Aging, Washington University, St. Louis, Missouri.; 2 Brown School of Social Work, Washington University, St. Louis, Missouri.; 3 Social System Design Lab, Washington University, St. Louis, Missouri.; 4 Faculty of Social Sciences, System Dynamics Group, University of Bergen, Norway.

**Keywords:** Caregiving, Productivity, Productive aging, Systems science, Volunteering, Working

## Abstract

Gerontologists have argued that the growing human capital of the aging population can be better marshaled as a resource for families, communities, and society at large. Additionally, this active, purposeful engagement can produce positive outcomes for older adults themselves. In this manuscript, we propose that existing conceptual frameworks articulating antecedents and outcomes of productive engagement, including working, volunteering, and caregiving can be improved using a system dynamics (SD) approach. Through a series of five unstructured group model-building sessions, experts from gerontology and systems science developed a qualitative SD model of the productive engagement of older adults. The model illustrates the reciprocal and dynamic nature of the stocks of human capital of older adults, social capital of older adults, and family resources; the engagement of older adults in productive activities; and the social and organizational variables that affect the flow and depletion of these stocks. Given this is the first attempt to develop a SD model for productive engagement in later life, the model is preliminary and heuristic. However, it offers a new approach to advancing theory and research on productive engagement in later life. Further, it can guide the development of mathematical models to estimate the effects of changes in any part of this system.

**Translational Significance**

System dynamics can illuminate dynamic, complex, and reciprocal relationships and guide theoretical and empirical understanding to increase productive engagement in later life.

In 1985, Robert Butler introduced the concept of *productive aging* to shift the focus from the dependency of older adults to their contributions to families and communities (Butler & Gleason, 1985). Since then, it has been widely noted that older adults are a vastly underutilized resource that society cannot afford to disregard, especially in the face of population aging (Rowe & Kahn, 1997). Over the last three decades, scholars have advanced a research agenda to increase our understanding about the productive engagement of older adults. Productive activities have been operationalized as any activity, paid or unpaid, that generates goods and services of economic value; and working, volunteering, and caregiving are usually the focus. Various conceptual models have been employed to identify antecedents and outcomes of such activities. Antecedents have included individual characteristics as well as social and environmental factors. The potential of policy and programs to maximize and support productive engagement is widely recognized. Outcomes of the productive engagement of older adults have been documented for individuals and families, and wider outcomes for society have been theorized.

A full understanding of the complex relationships between the antecedents and outcomes associated with the productive engagement of older adults as well as the barriers and facilitators to this engagement is important to maximize the productive potential of the older population. To date, most studies have been constrained by linear models and a focus on nonreciprocal relationships. Clearly, a phenomenon as multifaceted as productive engagement is more complex than current models suggest. The field of system dynamics (SD) offers a promising path to a more complete and nuanced understanding of the feedback structures between individuals, families, and society, as well as the outcomes of potential program and policy changes to increase opportunities for older adults to become productively engaged.

This manuscript is the result of the efforts of a transdisciplinary, transnational group of scholars at the Brown School of Social Work at Washington University in St. Louis who met over a 4-month period in late 2015 and early 2016, with support from the Friedman Center for Aging and the Social System Design Lab. Three full-time faculty, one doctoral student, and three international visiting scholars who are experts in social gerontology, demography, and SD formed a workgroup to explore the intersection of productive engagement in later life and SD. The purpose was heuristic, with the aim to employ an increasingly popular method for understanding complex social issues in the field of aging.

This manuscript begins with a brief review of the conceptualizations regarding productive engagement in later life and their empirical support. Next, we review the major tenets of SD and apply them to the essential elements of the existing productive engagement frameworks. We then offer an SD framework—specifically, a stock and flow diagram—that will guide theoretical and empirical development in the field of productive engagement. To conclude, we outline the process of theoretical development and empirical testing using SD, while discussing the implications of this work to explicate the potential of an aging society.

## Current Conceptualizations of Productive Engagement

Bass and Caro (1996, 2001) presented the first conceptual framework regarding the antecedents of productive engagement. This model focused on the influences of social policy (e.g., government and employer policies), environment (e.g., demographic changes), situation (e.g., socioeconomic status), and individual factors (e.g., motivation) on the level of participation in productive activities. Building on this framework, Sherraden, Morrow-Howell, Hinterlong, and Rozario (2001) articulated the antecedents to productive engagement in two domains: individual capacity (sociodemographic, health, and educational factors) and institutional capacity (organizational characteristics, policies, and programs). The framework implies that these two capacities are requisites for maximal engagement and that individual capacity will be underutilized until institutional capacity grows to engage it. Further, the model specified that outcomes of this engagement are multilevel, including individuals, families, communities, and society.

**Figure 1.**
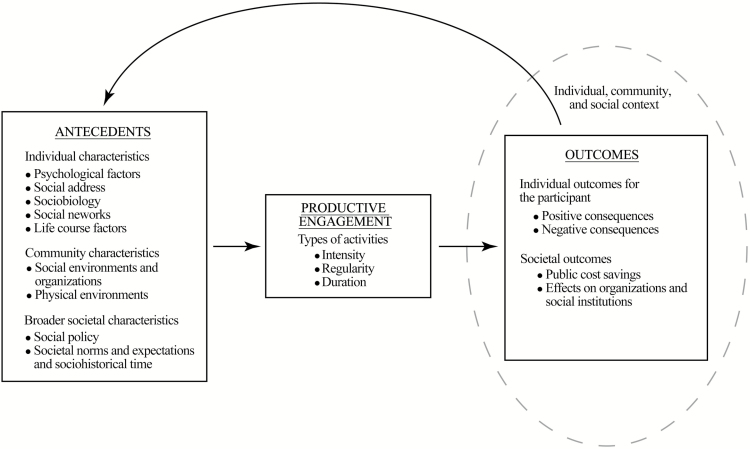
Conceptual framework guided by ecological systems theory on antecedents and consequences of productive engagement in later life. From Morrow-Howell, N. & Greenfield, E. (2016). Productive engagement in later life. In L. George & K. Ferraro (Eds.), *Handbook of Aging and the Social Sciences* (8 edn, pp. 293–309). London: Academic Press.

The conceptual framework shown in Figure 1 derives from previous models by adding specificity to the form of the productive activities, antecedents, and outcomes (Morrow-Howell & Greenfield, 2016). Antecedents are conceptualized as individual, community, and societal. The intensity, regularity, and duration of engagement in working, volunteering, and caregiving is posited to lead to both positive and negative outcomes for older adults; societal outcomes are specified as effects on organizations and institutions, as well as public costs.

Empirical work has demonstrated that factors at each of the levels identified in these models affect engagement. For example, education and health are key predictors of late-life employment and volunteering (Employment Benefit Research Institute, 2011; McNamara & Gonzales, 2011). Also, gender and ethnicity relate in various ways to all types of productive engagement. As examples, older Hispanic and African Americans are more likely to be employees and formal volunteers (Johnson & Park, 2011); older African Americans provide more care to nonrelatives than other groups (National Alliance for Caregiving & AARP, 2015); and rates of custodial grandparenting are higher among ethnic older adults than Whites (Livingston & Parker, 2010).

The effects of environmental contexts have been documented. For example, higher perceived availability of resources, such as amenities and services, traffic conditions, and safety conditions in neighborhoods, has been associated with an increased likelihood of volunteering by older adults (Dury et al., 2014). At the broader social level, organizational characteristics of employers, like job flexibility and perceived age discrimination in the workplace, affect older workers’ experiences and retirement decisions (Cahill, Giandrea, & Quinn, 2015; Roscigno, 2010). In sum, empirical work suggests that factors at all levels, from the individual to the environment, affect older adults’ engagement in productive activities.

There is also a substantial literature that documents the outcomes of productive engagement for older individuals. It has been demonstrated that work, in general, leads to more positive physical and mental health outcomes in later life (Staudinger, Finkelstein, Calvo, & Sivaramakrishnan, 2016). Volunteering also has been associated with positive health and psychological outcomes as well as higher odds of employment (Hong & Morrow-Howell, 2010; Kim & Ferraro, 2013; Spera, Ghertner, Nerino, & DiTommaso, 2013). Outcomes are not always positive: Caregiving can lead to increased stress, lower job satisfaction, poorer physical and mental health, and financial strain (Coughlin, 2010; Feinberg, Reinhard, Houser, & Choula, 2011).

In summary, previous conceptual frameworks have guided many studies on the antecedents and outcomes of productive engagement among older adults. However, scholars have noted that the associated relationships are more complicated and nuanced than depicted. For example, in describing the model shown in Figure 1, the authors stated, “For the sake of parsimony, we formulate linkages among the constructs as largely unidirectional, with antecedents leading to productive engagement and outcomes following productive engagement. We recognize that many of the embedded associations are likely bidirectional” (Morrow-Howell & Greenfield, 2016, p. 299). A compelling example of the limitations of the current modeling are the studies of working and volunteering in later life. Researchers on these topics have long recognized the problem of social selection versus social causation and the reciprocal nature of the relationship between these productive activities and health has been documented (Li & Ferraro, 2005; Thoits & Hewitt, 2001). This bidirectional association is usually modeled linearly, with health predicting productive engagement in work or volunteer roles or productive engagement predicting better health outcomes, although some research has demonstrated the bidirectional nature of the relationship via longitudinal designs (Wickrama, O’Neal, Kwag, & Lee, 2013). Yet, these conceptual frameworks do not allow for the more complicated reality to be studied: organizational arrangements affect the experience of working/volunteering, and these experiences affect health outcomes of the engagement, while changes in health relate to on-going to involvement in productive roles. To better model and test the complexity of this phenomenon, SD offers a more elaborate understanding of the feedback structures and leverage points.

## A New Perspective on Modeling Productive Engagement: System Dynamics

With its roots in feedback control theory in engineering, SD (e.g., Forrester, 1961; Sterman, 2000) is a method that aims to understand the behavior of a system as a result of how two or more feedback mechanisms interact with one another. SD has been widely applied to understand a variety of complex social problems, including domestic violence (Hovmand & Ford, 2009), infant mortality (Munar, Hovmand, Fleming, & Darmstadt, 2015), obesity (Fallah-Fini, Rahmandad, Huang, Bures, & Glass, 2014), prescription medication abuse and overdose deaths (Wakeland, Schmidt, Gilson, Haddox, & Webster, 2011), and smoking cessation (Tobias, Cavana, & Bloomfield, 2010), as well as more population-level concepts such as urban decline (Forrester, 1969) and the national economy (Forrester, 1980).

SD argues that feedback mechanisms or circular causality are central to social reality, providing a perspective that is distinct from more traditional statistical techniques that infer unidirectional cause and effect, blurring the distinction between antecedents and outcomes present in more traditional conceptual models. Although statistical methods like structural equation modeling (e.g., Bollen, 1989) can capture reciprocal effects, SD generally focuses on nonlinear feedback relationships that lead to shifts in the influence or dominance of feedback loops over time (Hovmand and Chalise, 2015). Consequently, SD is unique in its ability to describe and understand the complex dynamics of social systems.

SD uses both informal causal maps and formal mathematical models, often with computerized simulations, to test hypotheses about the relationship between the structure and dynamic behavior of a system from an endogenous, or feedback, perspective (Richardson, 2011). That is, the goal in SD is to examine how relationships between endogenous variables (e.g., programs serving older adults and the development of social capital) can explain system behavior (e.g., human capital outcomes over time, perhaps operationalized as physical and mental health), as opposed to relying on exogenous explanations of system behavior (e.g., the impact of the national economy on older adults).

SD uses a set of terms and diagramming conventions that may not be familiar to those new to the field. As such, we will now define and illustrate key terms diagramming conventions.

### Stocks

In SD, stocks (or “state variables”) are variables that accumulate or deplete over time and are represented by boxes in diagrams. Stocks describe the level of accumulation of that variable at any given point in time. To illustrate a common educational example in SD, stocks can be described as bathtubs with changing levels of water over time (e.g., Sterman, 2001). Another example of a stock is a savings account at a bank, in which the accumulated deposits and withdrawals over time are mathematically identical with the amount of money in the account. Accumulation, which is a function of different rates of inflow and outflow in a stock, is a characteristic of systems that is often overlooked in statistical models (Hovmand and Chalise, 2015).

### Flows and Rates

In SD models, flows are represented by the double lines or “pipes” in an SD model. Flows are not variables per se, but rather visual representations of the inflows and outflows that fill or drain a stock. Using the bathtub example, flows are the faucet and drains that serve to fill or drain the bathtub with water. The “valves,” or double triangles that appear like hourglasses, represent the rates of flows, which are variables that define the speed of accumulation or depletion of stocks. These rates can be understood as the knobs on the faucet and the aperture of the drain, which can be opened or closed to control the rates of inflow and outflow. In other words, whether the level of the water in a bathtub is increasing or decreasing is a function of the water flowing into the tub and draining from the tub. If the total inflow equals the total outflow, the level of the water remains constant and the system is said to be in dynamic equilibrium. If the total inflow is greater than the outflow, the water level rises. Conversely, if it is less than the outflow, the water level falls. Using the savings account example, flows are the various activities, such as deposits, interest, withdrawals, and fees that control the amount of money in the savings account. The relationship between rates of inflow and outflow in a savings account determine whether the savings account grows or shrinks over time.

Although we have just provided two simple examples of stocks and flows, there are underlying mathematics at work. A key point about the distinction between stocks and flows that is often misunderstood in social sciences is that the *only* mathematical way a stock can change is through the influence of a flow. For example, a savings account (a stock) increases with deposits and interest (inflows) and decreases with withdrawals and fees (outflows). How fast the savings account changes over time is a function of the net rate of change, that is, the sum of the deposits and interest minus the withdrawals and fees. Similarly, the only way that the level of the water in the bathtub (a stock) can increase or decrease is through one or more flows. In other words, *any variable* that influences a stock variable *must* have one or more flow variables as a mediator variable.

In SD (and more generally, in a system of ordinary differential equations), the *state of the system* or level of the stocks in a system at any given point in time *t* is the *accumulation* (in mathematics, the integral) of all the inflows and outflows over time up to that point in time *t.* For example, the current level of the water in the bathtub is the accumulation over time of all the water that has flowed into the tub through the inflows and all the water that has flowed out through the drain up to time *t*. Likewise, the amount of money in a savings account at time *t* is the accumulation of all the deposits and interest minus the withdrawals and fees.

### Auxiliary Variables

In principle, all equations in a dynamic model can be written as a function of stock and flow variables; however, this often obscures the logic of the hypothesized causal mechanisms forming a feedback loop. To address this, SD models typically include auxiliary variables that represent an intermediate calculation in the causal logic. Auxiliary variables are typically represented on their own without an icon. These are often used, for example, to represent the logic of decisions to increase or decrease flows that, in turn, directly affect the stock variables. Using the bathtub example, an auxiliary variable could be the cost of water, which might influence the amount of water you chose to use (the inflow). Using the savings account example (and ignoring the advances in mobile banking), an auxiliary variable could be the number of days per week the bank is opened, which might influence the number and amount of deposits and withdrawals on any given day of the week.

### Sources and Sinks

In addition to stock, flow, and auxiliary variables, SD diagramming explicitly call out the material and information boundaries of a system (called “sources” and “sinks”), shown as clouds. In the savings account example, money coming into the account through deposits and interest would typically be shown as flowing from a cloud into the savings account stock. In this case, the cloud (or source) represents a conceptual boundary of the system and assumes that there is an infinite supply of currency to flow into the savings account that is only limited by the interest and savings rate. If such an assumption is not true (e.g., there is a limited money supply, such as income from a short-term job), then the infinite source or sink should be replaced by a stock to model the inflows and outflows.

It is important to stress that both stock and flow variables can increase and decrease, can be used to represent both tangible quantities as well as intangible qualities, and should arguably be viewed as latent variables with underlying causal structures that may vary in how easy they are to measure. Ultimately, however, the main benefit of distinguishing stock variables from flow variables is in being able to formulate an endogenous theory of how a dynamical system is regulated by a set of reinforcing and balancing feedback mechanisms.

**Figure 2.**
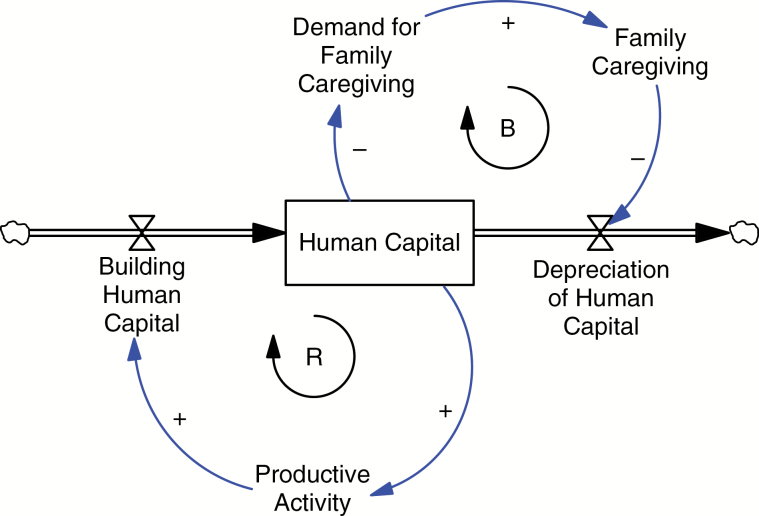
Stock and flow diagram of human capital. R = reinforcing feedback loop for increasing human capital through productive activity; B = balancing feedback loop for protecting human capital through family caregiving.

Figure 2 provides a simple example of a stock and flow diagram related to the productive engagement of older adults that uses the diagramming conventions described previously. It illustrates the inflow from *building human capital* (a rate of change) to *human capital* (a stock), as well as the outflow from *human capital* to *depreciating human capital* (another rate of change). In addition to stocks and flows, auxiliary variables (e.g., *productive activity* and *demand for family caregiving*) are shown. Hypothesized causal relationships involving auxiliary variables are represented by directed arcs with a sign representing the polarity of the association. For example, as *human capital* decreases, there is more demand for *family caregiving*. Unlike stock variables that change over time as a consequence of variable rates of inflow and outflow, auxiliary variables change instantly with their antecedent variables. For example, in Figure 2, *demand for family caregiving* increases as soon as *human capital* decreases. Lastly, all information outside of this particular system are represented by sources and sinks.

### Feedback Loops

Feedback mechanisms or loops are created as causal chains and can generally be divided into reinforcing and balancing feedback loops. Reinforcing (“positive”) feedback loops accelerate change in a variable. For example, an initial increase in the rate of *building human capital* adds to the stock of *human capital* faster, which in turn leads to an increase in *productive activity*, increasing the rate of *building human capital* even more, forming a reinforcing feedback loop. It is important to note that this reinforcing feedback loop (“R” in Figure 2) can behave as either a “virtuous cycle” where more human capital grows, or a “vicious cycle” where human capital dwindles. In other words, a decrease in *productive activity* decreases the rate of *building human capital*, which leads to decreasing stocks of *human capital*, further reducing *productive activity*.

The second type of feedback loop is a balancing (“negative”) feedback loop (“B” in Figure 2). Balancing loops counteract a change in a variable. In this example, an increase in *family caregiving* to older adults slows the rate of *depreciation of human capital*, which in turn helps retain older adults’ *human capital* above what it would have been had they not received care, which then lessens *demand for family caregiving*.

A key point in SD is that the dynamics of stocks are primarily driven by how a set of balancing and reinforcing loops interact with each other. For example, whether the stock of *human capital* in our example increases or decreases over time is related to which of the two feedback loops is most influential. In general, a mathematical consequence of nonlinear feedback systems is that while there can be many balancing and reinforcing feedback loops, typically only a small subset is driving or dominating the dynamics of a variable at any given time. This dominance tends to shift from one set of feedback loops to another over time. One major implication of this is that it can be difficult to correctly identify the leverage points in a nonlinear feedback system. Meadows (1999) provides a heuristic for identifying possible places to intervene in a system. For example, leverage points include strengthening or weakening both reinforcing and balancing feedback loops or changing the rules of the system (e.g., incentives or punishments).

This paper conceptualizes the interactive nature of productive engagement in later life on the individual, family, and society through SD. This conceptualization builds on current conceptual frameworks and empirical findings in the literature on productive engagement and introduces a conceptual stock and flow model. Future research will further specify concepts and relationships as well as test key assumptions through computer simulations, which are ultimately needed to identify high-impact leverage points (Harich, 2010) and guide intervention decisions.

## Group Model Building: Bringing Together Productive Engagement and System Dynamics

Group model building is an iterative, interactive method for engaging participants to create SD models (Luna-Reyes et al., 2006; Vennix, 1999). Group model building is a process that introduces the concepts of SD, including stocks, flows, and feedback loops; incorporates these concepts with the subject at hand (in this case, productive engagement in later life); leads to the development of more complex SD models that incorporate several stocks, feedback loops, and subsystems; and finally, uses the models developed to spur group discussion on system-level interventions (Richardson, 2013).

Group model building engages practitioners, scholars, and stakeholders who represent different disciplines and perspectives, incorporating knowledge from disparate fields into a shared project. In our case, seven scholars met over a period of 5 months in the Seminar on System Dynamics and Productive Engagement in Later Life. For each session, a series of readings on the history, theories, and conventions of both SD and productive aging, as well as a lead discussant, were assigned (see the Supplementary Materials for the seminar description and complete list of readings). Three of the participants were experts in productive engagement of older adults, two in SD, and two in demography. During discussions, participants drew a series of models—including linear and SD models—on the white board, steadily increasing in complexity with each session. By the third session, participants digitized these models in the Vensim software package. Throughout, participants discussed the underlying feedback structures that shape older adults’ productive activities, steadily increasing their use of SD terminology. Following the five sessions, participants attended a series of ad hoc meetings over the next 2 months to finalize the full model.

## Our Model: A New Perspective on Productive Engagement in Later Life

**Figure 3.**
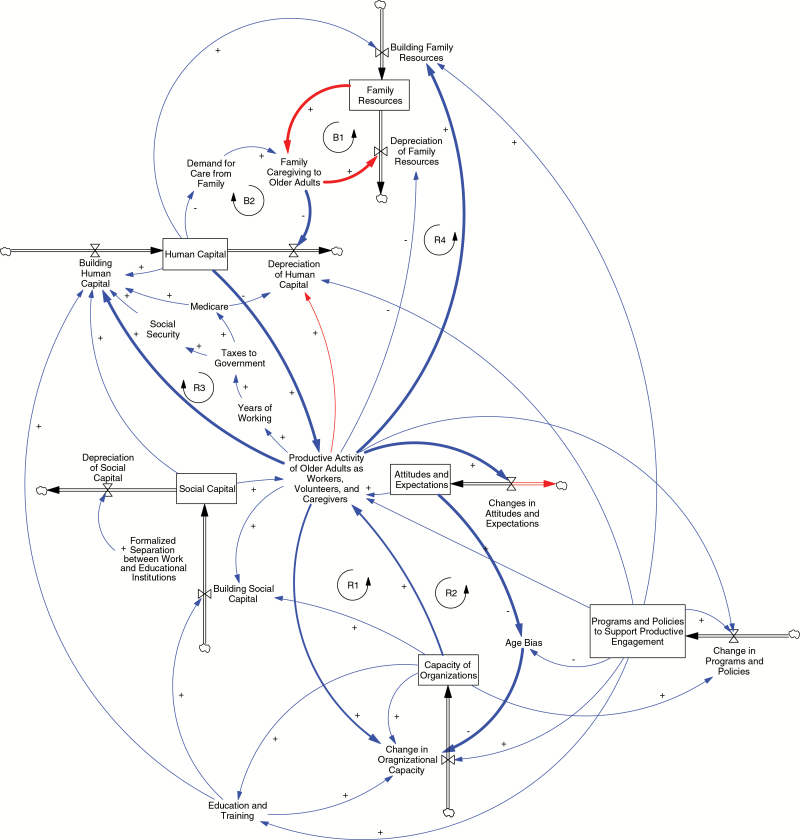
Stock and flow diagram of productive engagement in later life. This diagram contains many feedback loops, from simple to complex, and all cannot be identified in a simple and clear fashion. R1, R2, R3, and R4 are examples of reinforcing feedback loops and B1 and B2 are examples of balancing feedback loops. Full descriptions of each can be found in Table 1.

The results of the group model building include a stock and flow diagram of productive engagement in later life and a set of explanations regarding its concepts and relationships. As shown in Figure 3, at the center of the model is the concept of *productive activity of older adults as workers, volunteers, and caregivers* (“productive activity”). For this concept, we focus on paid and unpaid work, including working, volunteering, and caregiving. The model includes several stocks identified through the assigned readings and subsequent discussions, including human and social capital of older adults, family resources, capacity of organizations to fulfill their purposes, programs and policies to support productive engagement of older adults, and societal attitudes and expectations about older adults. These stocks are all part of a complex system of feedback loops that determine the level of productive activity of older adults.

The stock of older adults’ *human capital*, which includes health status, educational attainment, and financial status, is a naturally growing stock in the United States, given demographic shifts. However, the use of human capital is restricted by limited opportunity and support for productive engagement of older adults, which include ageist attitudes and expectations, limited programs and policies aimed at increasing engagement, and the low capacity of organizations to maximize the engagement of older participants. As such, older adults’ human capital can be viewed as underutilized.

The human capital of older adults can be better utilized by changing societal attitudes and expectations about older adults, increasing programs and policies to support productive engagement, and transforming organizations to maximally utilize this human capital. The *attitudes and expectations* stock illustrates that we can reduce age bias and change expectations about old age in this society, viewing older adults as experienced, competent, and committed to meaningful engagement—as opposed to current stereotypes of incompetent, useless, and “greedy geezers” (Lieberman, 2013). For example, it could become an expectation that older workers routinely receive coaching and training for career advancements, career switching, or new volunteer roles, instead of thinking about this as a low-return investment. The *programs and policies to support productive engagement* stock illustrates that we can enhance or expand the number of programs and policies to support older adults in paid and unpaid work. For example, we could expand the Senior Community Service Employment Program aimed at job training and placement for low-income older adults, or increase financial and psychosocial support for older caregivers through the expansion of consumer-directed care programs. The *capacity of organizations* stock illustrates that we can improve organizations’ abilities to maximize the involvement of older adults as workers, volunteers, students, and active citizens. For example, we can make workplaces more aging-friendly, improve age-neutral hiring and training processes, and offer more attractive and flexible volunteer positions (Halvorsen & Emerman, 2013). These factors that involve programs, policies, and organizations correspond to the institutional or organizational capacity concepts used in previous frameworks on productive engagement in later life (Sherraden et al., 2001).

In sum, this model suggests that there are modifiable conditions to increase the utilization of human capital in productive activities. The lower half of the model depicts the flow of older workers and volunteers into organizations, including businesses, nonprofit and public organizations, and educational institutions. As shown in Table 1, reinforcing feedback loop *R1* is illustrated by the arrow from *productive activity* to *change in organizational capacity*. This represents the flow of older adults into organizations, providing person-power and enabling organizations to better fulfill their missions, leading to the increased engagement of older adults in work and volunteer roles. Reinforcing feedback loop *R2* is illustrated by the arrow from *productive activity* to *changes in attitudes and expectations* about older workers and volunteers; ultimately, this may reduce age bias and increase organizational capacity by creating more supportive work environments for older adults—thus increasing productive activity.

**Table 1.** Exemplar Feedback Loops

Reinforcing Feedback Loops	
Title	R1. Building organizational capacity through productive activity
Loop	Capacity of Organizations → Productive Activity → Change in Organization Capacity → Capacity of Organizations
Explanation	An organization with higher capacity for engaging older adults can bring more older workers and volunteers into paid and unpaid work. This, in turn, leads to increased capacity for organizations to fulfill their missions.
Title	R2. Reducing age bias through productive activity
Loop	Capacity of Organizations → Productive Activity → Changes in Attitudes and Expectations → Attitudes and Expectations → Age Bias → Change in Organizational Capacity → Capacity of Organizations,
Explanation	An organization with higher capacity can engage more older adults. More older adults contributing to the organization could lead to changes in attitudes and expectations about older adults, thereby reducing aging bias. Reductions in age discrimination could further expand the capacity of organizations.
Title	R3. Building human capital through productive activity
Loop	Human Capital → Productive Activity → Building Human Capital → Human Capital
Explanation	When human capital of older adults is actively engaged in productive activity, the capital (finances, health, knowledge, etc.) can be further built or at least maintained, as opposed to depleted due to disengagement.
Title	R4. Building family resources through productive activity
Loop	Productive Activity → Building Family Resources → Family Resources → Family Caregiving to Older Adults → Depreciation of Human Capital → Human Capital → Productive Activity
Explanation	Productive activity, including working and caregiving by the older adults, can build family resources. Increased family resources can be utilized to provide assistance to the older adults when needed. This instrumental, financial, or emotional assistance by the family to the older adults can maintain or prevent depletion of the older adults’ human capital, enabling them to continue to be productively engaged.
Balancing Feedback Loops	
Title	B1. Depleting family resources through family caregiving
Loop	Family Resources → Family Caregiving to Older Adults → Depreciation in Family Resources → Family Resources
Explanation	Family resources, like time, health, and money, are depleted when family members provide assistance to older adults. With fewer resources, members provide less caregiving to older adults, reducing the amount of family resource depreciation.
Title	B2. Protecting human capital through family caregiving
Loop	Human Capital → Demand for Care from Family → Family Caregiving to Older Adults → Depreciation of Human Capital → Human Capital
Explanation	As the human capital of older adults depreciates (health or financial), there is an increased demand for caregiving and family members provide assistance. This assistance prevents the depletion of older adults’ capital, in that functioning is maintained (e.g., transportation is provided or finances are stabilized).

This model also depicts the process of building and depleting the stock of *human capital*. The human capital of health depletes as individuals reach the end of their lives, and too often in these extended years, financial capital also depletes. Yet, older adult human capital is depreciating more rapidly than it might due to the failure of current social structures to maintain and replenish it. Much of the effort of gerontologists has been to prevent this depletion (e.g., delaying disability, compressing morbidity, and preventing total financial decumulation), and this figure captures these ideas by focusing on the role of productive engagement through the continual building or replenishing of human capital. Reinforcing feedback loop *R3* illustrates that *productive activity* leads to *building human capital*, which increases the stock of *human capital*, further increasing *productive activity*. In sum, active engagement can lead to better health, increased education, and increased financial security. Again, this reinforcing feedback loop is not as vital as it could be due to of the current constraints on productive engagement.

A similar story is depicted about the building and depletion of the stock of *social capital* in older adults. Currently, social capital is depleted when older adults separate from work and education institutions or reduce their community participation. This depletion of social capital reduces productive engagement because social capital—for example, a professional network—can lead to paid and unpaid work. However, a reinforcing feedback loop can be created if older adults are productively engaged, thereby building social capital.

The engagement of older adults in productive activities can also have negative effects on the human capital of older adults, represented by the red link from *productive activity* to *depreciation of human capital*. For example, working longer in certain employment conditions can reduce health and mental health, and the negative effects of caregiving on older adults are widely documented. Additionally, there is link between *productive activity* and *depreciation of family resources*, capturing how older adults’ productive activities can compete for time and energy. For example, working older adults may not be able to provide caregiving services to the family. The model also shows how policies to support productive engagement can mitigate the negative effects on the rate of human capital depletion. Currently, these efforts are perhaps best represented by caregiver and grandparent support programs.

The arrow from *productive activity* to *building family resources* represents the direct contribution by older adults to family resources. For example, if older adults work longer and achieve higher levels of economic security, they can contribute to family finances. Further, if older adults engage in the productive activity of caregiving, including grandparenting and spousal/relative care, other family members can focus more on their jobs and other responsibilities. In other words, via *productive activity*, older adults can contribute to the stock of *family resources* rather than drain it. Higher levels of *family resources*, then, lead to an increased ability to provide *family caregiving to older adults* when they need it. This, in turn, protects older adults’ human capital (i.e., decreases the rate of *depreciation of human capital*), which leads to the maintenance of *productive activity*. This is illustrated by reinforcing feedback loop *R4*.

There are several subsystems in the larger model that can be detailed. For example, a subsystem that is related to the productive activity of paid work represents the claim in the literature that working longer will have positive effects at the societal level, reducing demands of population aging on entitlement programs (Ellis, Munnell, & Eschtruth, 2014). If we change attitudes, expectations, programs, and policies to support older adults as employees, the years of paid employment may be extended. Through longer working lives, older adults will pay Federal Insurance Contributions Act (FICA) taxes longer, supporting the Social Security and the Medicare systems. These more sustainable public insurance systems lead back to increased human capital of older adults (e.g., improved or maintained personal finances and health) which, ultimately, leads to increased or continued engagement. This is an example of a reinforcing feedback loop.

There is another subsystem that regards family caregiving to older adults with functional and financial limitations. Family resources—a stock of physical, psychological, and financial assets—are often depleted when older family members require assistance due to the depletion of their human capital. Balancing feedback loop *B1* illustrates how *family caregiving to older adults* leads to the *depreciation of family resources*. As families provide caregiving assistance to older adults, their resources are depleted, leaving a smaller stock of *family resources* from which to provide additional caregiving. This loop is balanced because as there are fewer family resources to use for caregiving of older adults, there are also fewer resources to be depleted. As depicted in balancing feedback loop *B2*, the provision of *family caregiving to older adults* slows the *depreciation of human capital* in older adult care recipients, serving as a protective factor. This feedback loop may also be true for older adults with dwindling finances who need financial support from family members to maintain certain conditions. On the other hand, if the stock of *human capital* of older adults is not diminished—if they remain physically, mentally, and financially healthier—there will not be as much *demand for care from family*. As discussed earlier, if older adults’ human capital is maintained, there can be a flow of assistance in the opposite direction, with older adults assisting their children, grandchildren, spouses, and other family and friends. This would lead to a far healthier society on several measurements.

## Contributions of the New Model

This manuscript describes the development of a dynamic and complex conceptual model of the productive engagement of older adults, building on frameworks that have come before. We aimed to demonstrate the effects of multiple and interlocking factors associated with engaging older adults as workers, volunteers, and caregivers. The model illustrates how changes in one factor can have wide-ranging and reciprocal impacts on other factors. We believe that the model illuminates more clearly than previous models several key points about the productive engagement of older adults.

First, scholars studying productive engagement have long argued that the level of participation by older adults in paid and unpaid work can be most effectively influenced by extra-individual factors like programs, policies, and organizations. This model visually highlights the prominent and widely reciprocal effects of factors outside the individual attributes of older people. For example, this model depicts the system-wide effects of expanding programs that engage older volunteers (e.g., Experience Corps and Foster Grandparents) or increasing the capacity of employers to attract and retain older workers. Previous models seem to include individual factors and extra-individual factors more symmetrically and do not call out as clearly the fundamental assumptions about the primary role of organizational and policy arrangements in promoting productive engagement.

Second, a major theme in the productive engagement literature has been the “win–win” situation represented by the positive individual, family, and societal effects of increased numbers of older adults in paid and unpaid work. This model spotlights that increasing the flow of human capital into productive activities is essential to achieve these multiple benefits. Positive effects on society are achieved by increasing the number of experienced workers and volunteers flowing into organizations, the extension of working years that affect public entitlement programs, and the number of older caregivers who aid family members. Positive effects on older individuals are achieved by the reciprocal relationship between productive activities and building or replenishing human and social capital. Thus, this model offers increased clarity in operationalizing this “win–win” scenario while also explicating the negative effects of productive engagement, such as from caregiving or working in undesirable circumstances. As such, this model can be used to explicitly identify and illustrate the unintended consequences of changes to the system.

Finally, the frequent discussions among gerontologists about the compression of morbidity, delaying of disability, prevention of financial decumulation, and creation of a “third age” all refer to the extension of human capital further into the life course. Indeed, Alvor Svanborg (2001) suggested that the biggest dividend of productive engagement would come from postponing decline associated with aging. The central role of human capital—both in building and depleting it—is clear in this model. Going forward, SD offers both theoretical and analytical means to guide program and policy developments aimed at maintaining and using the human capital of the older population.

## Going Forward

In this paper, we have presented an initial and conceptual SD model that underlies the individual-, family-, and societal-level interrelationships of productive engagement in later life. As such, this conceptual model represents an early stage of theory specification (e.g., Meehl, 1990) that is the start of an iterative program of theory development to be undertaken through modeling and empirical testing, all within a progressive program of research (Lakatos, 1970; Ostrom, 2005). Figure 3 represents a first and necessary step in developing a more complex, accurate, and testable model of productive engagement in later life through the lens of SD. The process of improving the model is iterative, in which we first lay out our assumptions and qualitatively test the logic of the model. Going further, we can apply quantitative parameter values—such as estimates of initial conditions and rates of change that determine flows—drawn from the existing literature and extant data. The ultimate goal of SD modeling and simulation is to more accurately articulate theory while empirically testing leverage points for future interventions and social change. The following two sections discuss in more detail the potential next steps for theory development and empirical development using SD.

## Theory Development

The goal of theory development is to increase confidence that the SD model reflects the actual structure of the system. Once a preliminary conceptual model is developed (such as in Figure 3), we can then build confidence in the model by identifying errors and omissions through an iterative process of qualitative review using the most current literature. This iterative process can be enhanced through the development a simulation model based on this qualitative structure.

SD provides a set of meta-theoretical rules for formulating causal relationships in a similar way as multivariate regression analysis provides a set of meta-theoretical rules for formulating statements about the associations among variables. The chief difference is that SD models focus on feedback relationships that are represented as a system of coupled and ordinary differential equations (Forrester, 1961). In other words, SD relies heavily on calculus-based mathematics instead of the more traditional statistics-based mathematics used in the social sciences. The iterative process of model specification begins in a manner similar to specifying “priors” (initial values and conditions) in Bayesian statistics, in which an initial, quantified, and simulatable model is tested to establish the internal logic and validity, as well as to check for gaps in the reasoning. Essentially, we are trying to ground the model in rough estimates from empirical data and the literature.

Consider a simple reinforcing feedback loop between the stock of *social capital* and *productive activity* (shown in Figure 3). *Productive activity*—which we will operationalize as volunteering for this example—has been shown to increase the number of friends (Morrow-Howell, 2008). This, in turn, increases one’s stock of *social capital*, which might be operationalized as the size of friendship networks. To complete this feedback loop, having higher levels of *social capital* have been shown to lead to higher levels of volunteering (i.e., the variable *productive activity*; McNamara & Gonzales, 2011). Estimates of the effect of friendship network size on levels of volunteering could be used to provide rough estimates for early versions of the quantified simulation models. The process of building a coherent model in which the units of these stocks, flows, and auxiliary variables are consistent and the effects are both logically and mathematically sound occurs through error identification and correction. A basic test of model structure asks whether the quantified simulation model will allow us to consider whether the structure of the system and parameter estimates can reproduce past behavior in the system. Through running multiple iterations and refinements, revisions to the structure allow scholars to develop new understanding how and at what rate *social capital* may grow and depreciate. Adding complexity to the model, then, we could further explore the link between the *capacity of organizations* and the development of *social capital*, and so on.

Even though this process uses extant data, it is important to stress that this work focuses primarily on theory development. A simulation model that can replicate the empirical patterns, while encouraging, is generally considered a “weak” test, given the number of variables, flexibility of the mathematical forms relating to the variables, and the parameters and initial conditions that can be adjusted to fit the data. Further, using tests of statistical significance to test SD models is problematic, as the ultimate goal of the model-building process is to *fail* to reject the null hypothesis that our model replicates the real world (Barlas, 1996). However, one often can rule out a number of theories that seemed plausible through verbal reasoning and grounding in the published studies, due to the fact that these models could not generate the observed patterns of behavior.

Part of the challenge of confidence building in SD models is that the very nature of complexity makes it hard to draw logically valid inferences about the nonlinear relationships between variables that involve accumulations, delays, and feedback (Sterman, 2000). Face validity and replications of empirical trends are not sufficient to build confidence that a model is an adequate representation of the structure of a system. An example includes how *education and training* influence the rate of *building human capital* in our model while accounting for factors in other subsystems that have dynamic relationships with these variables. Although simple linear relationships between factors may be evident, human capital appreciation is embedded in and influenced by multiple feedback loops, such as *productive activity* and the development and depreciation of *social capital*. These loops may interact with *education and training* in a way that cannot be inferred through simple linear relationships. Thus, the result of using SD modeling with simulation is a formal verification of the logical consistency of a core theory around which one can build a progressive program of research.

## Empirical Development

Having formally verified a theory in terms of its logical consistency is valuable but can be pushed further through the development and testing of hypotheses through simulation. A simulation model might lead to a specific hypothesis about the influence of feedback mechanisms that ultimately lead to a final result. For example, a series of simulations could test the hypothesis that programs and policies to support productive engagement of older adults as workers and volunteers do more to reduce age bias in the workplace than programs and policies that directly target the age bias of younger colleagues. Simulations of this sort could lead to the identification of stronger leverage points to reduce age bias.

In many cases, the data needed for SD models already exist and come from observational and prospective studies, natural experiences, and re-analyzing results from systematic reviews and meta-analyses. Extant trends, associations, and prevalence estimates from secondary data and published studies—such as from the Health and Retirement Study and its sister studies, MIDUS, the Current Population Survey, and others—can be used to test, calibrate, and further refine this simulation model and examine potential leverage points.

A well-established experience from SD is that no single source of quantitative data contains all of the information needed to build a model and run a series of simulations regarding the productive engagement of older adults, or any other complex system (Forrester, 1992). Instead, this process involves utilizing estimates from the various data sources that exist and, when needed, collecting or estimating new data points. For example, a hypothesis may exist where conditions have been observed but the data have not been collected (e.g., the extent to which an organization has the capacity to offer education and training to its older workers and its potential effects on building participants’ human and social capital). Or, a hypothesis may exist about the relative relationship between two variables under a condition that has not yet been observed (e.g., the effect of offering a sabbatical or “gap year” for mid- to later-career workers on their total years of working). This type of simulation modeling would enable scholars to test hypotheses that cannot be currently tested in the real world due to time, resource, or other natural constraints. Furthermore, simulation models can also reveal the absence of important data, which can then be generated through primary research efforts and used to test the model or enhance the robustness of its specification.

Finally, SD models can also be used to design pilot studies to explore novel hypotheses in the real world. For example, one might use a formal simulation model to discover and develop policy interventions that increase human capital through productive engagement in later life, and then test these interventions in a pilot study while paying close attention to the intermediate mechanisms and predictors of outcomes suggested in the simulation model. The model could thus serve as a guide for the design and implementation strategy for the pilot study, as well as a framework for understanding both the outcomes and the specific inflection points and mechanisms through which an outcome failed to be met.

## Conclusion

SD can help to identify and assess the effects of changes at key leverage points. SD models could then estimate the short, intermediate, and long-term ramifications within the larger system, including changes in organizational capacity, human capital of older adults, demand for caregiving, and attitudes and expectations about older adults. Our model, created through a synthesis of the fields of productive engagement in later life and SD, suggests that these and other high-impact, system-level leverage points may exist. Future simulations—following the iterative procedures outlined in this report—will determine when, and under what conditions, these changes make a meaningful difference.

## Supplementary Material

Supplementary data are available at *Innovation in Aging* online.

## Conflict of Interest

None reported.

## Supplementary Material

Supplementary Materials
