# Supplementary material for: Conceptualizing Productive Engagement in a System Dynamics Framework
Source: Innov Aging. 2017 Sep 30;1(1):igx018. doi: 10.1093/geroni/igx018 (PMC6177040; doi:10.1093/geroni/igx018)
Supplement: Supplementary Materials [file igx018_suppl_supplementary_materials.docx]

Supplementary Materials


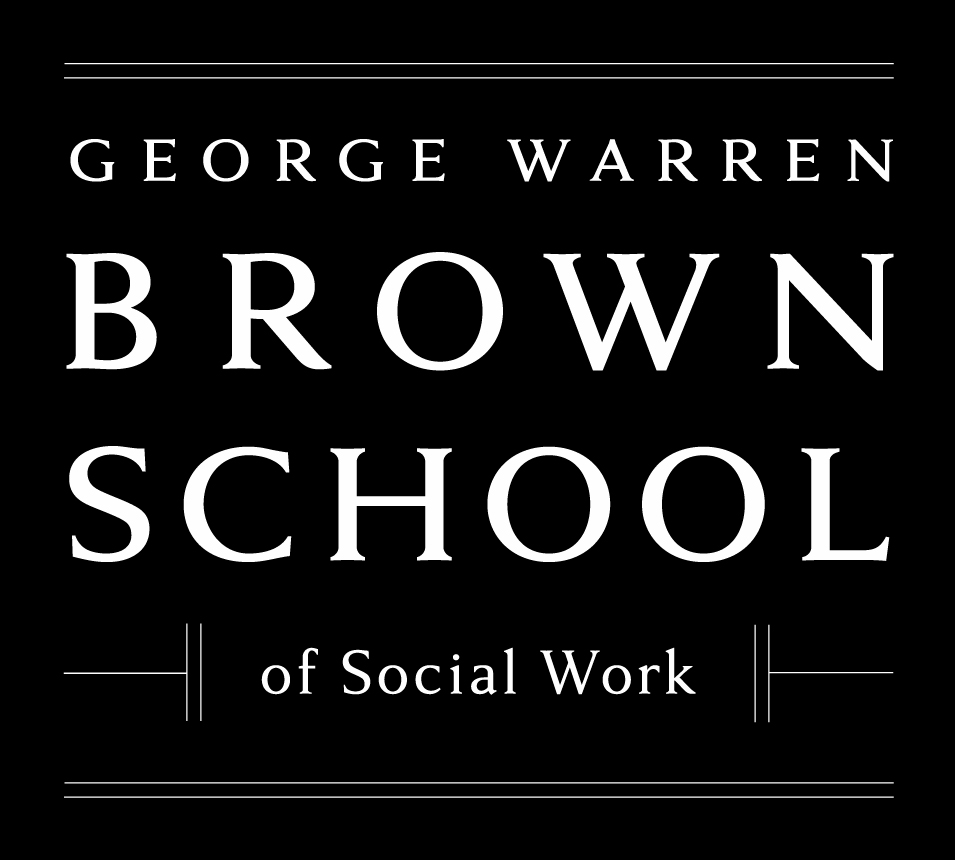


GEORGE WARREN BROWN SCHOOL OF SOCIAL WORK

WASHINGTON UNIVERSITY IN ST. LOUIS

**Seminar in System Dynamics and Productive Engagement in Later Life**

**Fall 2015**

# Seminar Description

This seminar provides an opportunity for doctoral students, visiting scholars, and faculty to develop a theoretical and methodological orientation to the productive engagement of older adults from a system dynamics perspective. The course covers the histories and key concepts and knowledge areas in both system dynamics and productive aging. Readings on the principles, history, and debates within the field of system dynamics, understanding and developing social theories from a system dynamics perspective, and methodological issues such as using data to develop models, research design, and confidence building tests are included. Further, key readings on the history, development, and current knowledge base of productive engagement in later life are included. This seminar prepares doctoral students for comprehensive and qualifying exams and the preparation of dissertation proposals, as well as all participants to begin to understand and apply a major method of systems thinking and research to their field of interest.

This course meets for 90 minutes per week and consists primarily of participant-led discussions on the readings. Further, we will engage in group model building in each meeting.

*Note: This supplemental document, made available to* Innovation in Aging *readers, is* a *condensed syllabus and complete reading list from this Fall 2015 seminar.*

# Course Outline and Readings

| Class 1 | **Introduction to System Dynamics and Productive Engagement in Later Life**   - Richardson, G. P. (2013). Concept models in group model building. *System Dynamics Review, 29*, 42-55. doi:10.1002/sdr.1487 - Morrow-Howell, N., & Wang, Y. (2013). Productive Engagement of Older Adults: Elements of a Cross-Cultural Research Agenda. *Ageing International, 38*(2), 159-170. doi:10.1007/s12126-012-9165-0 |
| --- | --- |
| Class 2 | **History and Foundations**   - Richardson, G. P. (2011). Reflections on the foundations of system dynamics. *System Dynamics Review, 27*(3), 219-243. doi:10.1002/sdr.462 - Forrester, J. W. (2007). System dynamics-a personal view of the first fifty years. *System Dynamics Review, 23*(2-3), 345-358. doi:10.1002/sdr.382 - Forrester, J. W. (2007). System dynamics-the next fifty years. *System Dynamics Review, 23*(2-3), 359-370. doi:10.1002/sdr.381 - Morrow-Howell, N., & Greenfield, E. A. (2016). Productive engagement in later life. In L. K. George & K. F. Ferraro (Eds.), *Handbook of Aging and the Social Sciences, 8^th^ Ed.* (pp. 293-313). London: Academic Press. - Bass, S. A., Caro, F. G., & Chen, Y. (1993). Introduction: Achieving a productive aging society. In F. G. Caro, S. A. Bass, & Y. Chen (Eds.), *Achieving a Productive Aging Society* (pp. 3-25). Westport, CT: Auburn House. |
| Class 3 | **Feedback Thinking**   - Richardson, G. P. (1991). *Feedback Thought in Social Science and Systems Theory.* Waltham, MA: Pegasus Communications, Inc. Chapters 1-3, 5-6 - Anderson, N. D., Damianakis, T., Kröger, E., Wagner, L. M., Dawson, D. R., Binns, M. A.,…The BRAVO Team. (2014). The benefits associated with volunteering among seniors: A critical review and recommendations for future research. *Psychological Bulletin*, *140*(6), 1505-1533. doi:10.1037/a0037610 |
| Class 4 | **Operational Thinking**   - Olaya, C. (2012). *Models that include cows: the significance of operational thinking.* Paper presented at the 30th International Conference of the System Dynamics Society, St. Gallen, Switzerland. https://www.systemdynamics.org/conferences/2012/proceed/papers/P1375.pdf - Meehl, P. E. (1990). Appraising and amending theories: The strategy of Lakatosian defense and two principles that warrant it. *Psychological Inquiry, 1*(2), 108-141. doi:10.1207/s15327965pli0102_1 - Hinterlong, J. E., Morrow-Howell, N., & Rozario, P. A. (2007). Productive engagement and late life physical and mental health: Findings from a nationally representative panel study. *Research on Aging*, *29*(4), 348-370. doi:10.1177/0164027507300806 |
| Class 5 | **Social Theory**   - Lane, D. C. (2001). Rerum cognoscere causes: Part I-how do the ideas of system dynamics relate to traditional social theories and the voluntarism/determinism debate? *System Dynamics Review, 17*, 97-118. doi:10.1002/sdr.209 - Lane, D. C. (2001). Rerum cognoscere causas: Part II--Opportunities generated by the agency/structure debate and suggestions for clarifying the social theoretic position of system dynamics. *System Dynamics Review, 17*(4), 293-309. doi:10.1002/sdr.221 - Sherraden, M., Morrow-Howell, N., Hinterlong, J., & Rozario, P. (2001). Productive aging: Theoretical choices and directions. In N. Morrow-Howell, J. Hinterlong, & M. Sherraden (Eds.), *Productive Aging: Concepts and Challenges* (pp. 260-284). Baltimore: The Johns Hopkins University Press. |
